# Development of Large-Scale Functional Brain Networks in Children

**DOI:** 10.1371/journal.pbio.1000157

**Published:** 2009-07-21

**Authors:** Kaustubh Supekar, Mark Musen, Vinod Menon

**Affiliations:** 1 Graduate Program in Biomedical Informatics, Stanford University School of Medicine, Stanford, California, United States of America; 2 Center for Biomedical Informatics Research, Stanford University School of Medicine, Stanford, California, United States of America; 3 Program in Neuroscience, Stanford University School of Medicine, Stanford, California, United States of America; 4 Department of Psychiatry and Behavioral Sciences, Stanford University School of Medicine, Stanford, California, United States of America; University College London, United Kingdom

## Abstract

The ontogeny of large-scale functional organization of the human brain is not well understood. Here we use network analysis of intrinsic functional connectivity to characterize the organization of brain networks in 23 children (ages 7–9 y) and 22 young-adults (ages 19–22 y). Comparison of network properties, including path-length, clustering-coefficient, hierarchy, and regional connectivity, revealed that although children and young-adults' brains have similar “small-world” organization at the global level, they differ significantly in hierarchical organization and interregional connectivity. We found that subcortical areas were more strongly connected with primary sensory, association, and paralimbic areas in children, whereas young-adults showed stronger cortico-cortical connectivity between paralimbic, limbic, and association areas. Further, combined analysis of functional connectivity with wiring distance measures derived from white-matter fiber tracking revealed that the development of large-scale brain networks is characterized by weakening of short-range functional connectivity and strengthening of long-range functional connectivity. Importantly, our findings show that the dynamic process of over-connectivity followed by pruning, which rewires connectivity at the neuronal level, also operates at the systems level, helping to reconfigure and rebalance subcortical and paralimbic connectivity in the developing brain. Our study demonstrates the usefulness of network analysis of brain connectivity to elucidate key principles underlying functional brain maturation, paving the way for novel studies of disrupted brain connectivity in neurodevelopmental disorders such as autism.

## Abstract 2

Large-scale rewiring of brain circuits in children leads to emergence of hierarchical organization in the mature adult brain.

## Author Summary

The disruption of normal brain organization in humans is believed to underlie a number of behavioral conditions, such as autism spectrum disorders (ASD) and attention-deficit/hyperactivity disorder (ADHD). To gain insight into how normal brain organization develops, we mapped functional brain connectivity in children and young adults, and used a network analysis to characterize and compare the organization of brain networks. Comparison of network properties revealed that while children and young adults' brains have similar organization at the global level, there were several key differences in connectivity. For example, children's brains had less of a hierarchical organization than young-adults. Most importantly, we show that the dynamic process of over-connectivity followed by pruning, which rewires connectivity at the neuronal level, also operates at the systems level, reconfiguring and rebalancing subcortical and paralimbic connectivity in the developing brain. Our findings demonstrate the utility of using network analyses of multimodal brain connectivity to study maturation of brain circuits, and suggest new avenues for future research on neurodevelopmental disorders such as ASD and ADHD.

## Introduction

Understanding the development of human brain organization is critical for gaining insight into brain organization and functions in adulthood as well as for investigating disorders such as autism spectrum disorders (ASD) and attention-deficit/hyperactivity disorder (ADHD), where normal developmental processes are disrupted. Neuroimaging studies of development have primarily focused on structural changes from childhood, to adolescence, and into adulthood. These studies have reported age-related changes in (1) overall brain volumes [1],[2], (2) volumes of individual brain areas [3],[4], (3) regional cortical thickness [5],[6], as well as (4) regional and global grey-matter and white-matter densities [7]–[9]. Collectively these studies have suggested that the human brain undergoes vast developmental changes in grey and white matter structure between childhood and adulthood. These changes are thought to reflect synaptic pruning and myelination observed at the neuronal level [8],[9]. More recently, diffusion tensor imaging (DTI) studies investigating the development of white-matter pathways have shown increase in anisotropy [10]–[12], decrease in overall diffusion [13], and maturation in major white-matter fiber tracts [14]–[19], with age. In spite of growing evidence from these studies for patterned brain development, the functional organization of the human brain in childhood is not well understood and it is also not clear how the above structural changes translate to differences in functional brain organization between children and adults.

Task-free (resting-state) functional connectivity MRI is a useful technique for investigating the functional organization of the human brain. This method detects interregional correlations in spontaneous blood oxygen level-dependent (BOLD) signal fluctuations [20],[21], and has been used to investigate brain networks involved in motor [20], sensory [22], attention [23], salience and cognitive control [24], and memory [25],[26] systems. However, only a small number of studies have examined developmental changes in functional brain organization. A few recent studies have examined developmental changes in functional connectivity of brain regions involved in attention and cognitive control [27] and the default mode network (DMN) [28], as well as in functional connectivity of anatomical structures such as the anterior cingulate cortex [29]. To our knowledge, the developmental changes in the functional organization of large-scale networks at the whole-brain level have not yet been investigated.

Here we use a graph theoretical approach to examine developmental changes in the large-scale functional organization of the human brain. Graph metrics such as the clustering coefficient and the characteristic path length [30],[31] have been shown to be useful measures of organization of large-scale networks. Briefly, graphs are data structures that have nodes and edges between the nodes [32]. The clustering coefficient is a measure of local network connectivity. A network with a high average clustering coefficient is characterized by densely connected local clusters. The characteristic path length is a measure of how well a network is connected. A network with a low characteristic path length is characterized by short distances between any two nodes. Many biological systems have small-world network properties, characterized by a high clustering coefficient and a low characteristic path length [30],[33].

These graph-theoretic metrics have also proven useful in modeling the large-scale functional and structural organization of the human brain [34]–[37]. In a graphical representation of a brain network, a node corresponds to a brain region while an edge corresponds to the functional connectivity between two brain regions. Functional connectivity networks of the human brain derived from electroencephalograms (EEGs), magnetoencephalograms (MEGs), and task-free functional magnetic resonance imaging (fMRI) data have been shown to exhibit small-world characteristics [35],[38],[39]. These studies suggest that small-world metrics are suited to quantify the global topological properties of large-scale organization of the human brain. Recently, in addition to small-world metrics, Bassett and colleagues used graph theoretic metrics such as hierarchy to characterize local topological properties of large-scale organization of the human brain. Using structural brain imaging data and modeling of interregional covariance in cortical thickness, they reported that hierarchical organization in anatomical human brain networks is characterized by the presence of frontal hubs [40]. A recent study of aging by Meunier and colleagues investigated the modular organization of large-scale functional brain networks using Newman's graph-based modularity metric. They reported that while both young and older adults showed modularity of network organization, the topological roles of the specific brain regions as well as the intermodular connectivity was significantly different between the two groups [41]. The use of small-world metrics along with more advanced graph theoretic metrics to characterize local organization of complex networks provides a new approach for investigating large-scale functional organization of the human brain at multiple levels of granularity.

We investigated developmental changes in the functional organization of large-scale brain networks at multiple levels by (1) creating whole-brain functional connectivity networks from task-free fMRI data, (2) characterizing the organization of these networks using metrics of global and local brain organization (including small-worldness and hierarchy, as defined in the Methods section), and (3) comparing these metrics of global and local brain organization between healthy children (ages 7–9 y) and young-adults (ages 19–22 y). In older adults (age ±40 y) it is now well established that large-scale brain networks have a small-world architecture that reflects a robust and efficient, nonrandom, functional organization [34],[35],[39]. Whether children and younger adults have a similar functional brain organization is currently not known. This question is important from a developmental perspective because the brain undergoes vast changes in structural connectivity during adolescence [9]. We hypothesized that the global functional organization of brain networks would be characterized by nonrandom, efficient, small-world characteristics in both subject groups, but that young-adults would show higher small-worldness compared to children, on the basis of previous neurobiological studies in humans and animals suggesting that developmental changes improve efficiency of information processing in the brain [42]–[44]. We further predicted that local organization patterns would be significantly different in children, reflecting a process of continuing structural maturation during the period between childhood and young adolescence. To further characterize developmental changes in the global and local functional organization of brain networks, we used the parcellation scheme of Mesulam [45] to examine functional organization in five key subdivisions: primary sensory, subcortical, limbic, paralimbic, and association areas. Additionally, developmental changes in the connectivity between these subdivisions (hereafter referred to as interregional connectivity in the manuscript) were examined. Lastly, to characterize the underlying developmental processes that produce these changes in the global and local functional organization of large-scale brain networks, we examined changes in functional connectivity as a function of DTI-based wiring distance between distinct brain regions. The formation of brain networks during development is thought to arise from a dual process of integration and segregation [27],[46]–[49]. Accordingly, we investigated whether there is in vivo developmental evidence for the emergence of functional segregation and integration in large-scale brain networks.

## Results

### Participants

Demographic and cognitive profile data for the child and young-adult groups are shown in Table 1. The two groups were well-matched and did not differ in IQ (*p* = 0.93) or gender (*p* = 0.75).

**Table 1:** Participant characteristics.

Measure	Children (*n* = 23)	Young-adults (*n* = 22)
Age	7.95^a^ (range: 7–9)	20.40^a^ (range: 19–22)
Gender	10 males, 13 females	11 males, 11 females
IQ	112 (range: 88–137)	112 (range: 97–137)
Years of education	2.52^a^ (range: 2–3)	14.5^a^ (range: 13–16)

Age and years of education, but not IQ nor gender, are significantly different in young-adults compared with children.

a Denotes significant differences between groups.

### Analyses of Small-World Metrics at Different Frequency Scales

**Figure 1:**
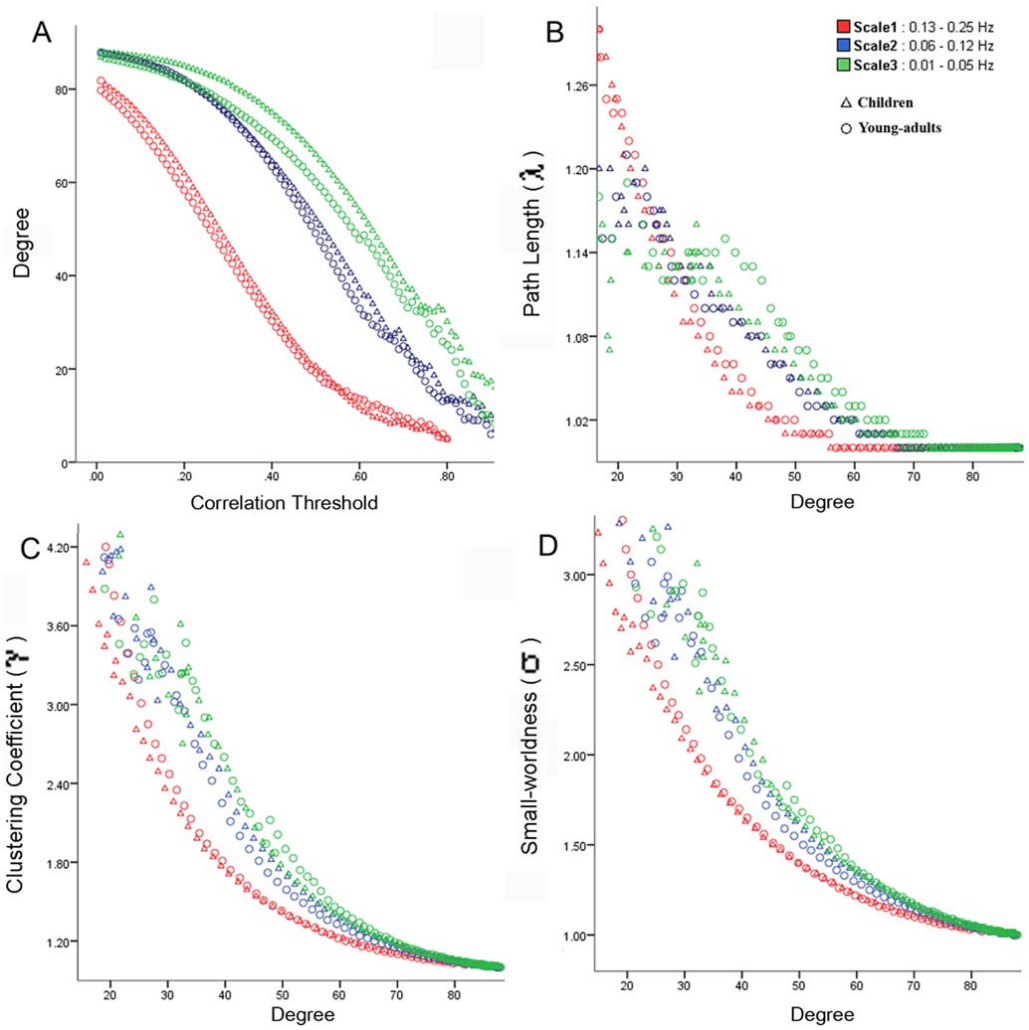
Developmental changes in whole-brain functional connectivity network metrics. Graph metrics: degree, path length (λ), clustering coefficient (γ), small-worldness (σ), for children (Δ) and young-adults (○) at three frequency intervals. (A) For both groups, the mean degree, a measure of network connectivity, is highest at scale 3 for a wide range of correlation thresholds (0.01<*R*<0.8). (B) The mean characteristic path length (λ) is low (1<λ<1.57) and shows similar trends at all the scales. (C) The clustering coefficient (γ) for both groups is highest at scale 3. (D) Due to higher mean γ values, the small-world measure σ (γ/λ) is highest at scale 3 for both groups. σ showed a linear increase in small-worldness as the threshold increased and the degree decreased. σ values for higher correlation thresholds are hard to interpret as at higher threshold values graphs of functional brain networks have fewer edges (smaller degree) and tend to split into isolated subgraphs. At each of the three scales, no significant differences in the degree, path length, clustering coefficient, and small-worldness values, for a range of correlation thresholds, were observed between children and young-adults. Scale 1 (0.13–0.25 Hz) is shown in red, scale 2 (0.06–0.12 Hz) is in blue, and scale 3 (0.01–0.05 Hz) is in green.

We first examined graph-theoretic metrics obtained for the functional brain networks constructed by thresholding (threshold values ranged from 0.01 to 0.99, with an increment of 0.01) the wavelet correlation matrix at three different frequency scales. Scale 1 encompassed 0.13–0.25 Hz, scale 2 encompassed 0.06–0.12 Hz, and scale 3 encompassed 0.01–0.05 Hz. As shown in Figure 1, for both the children and young-adult groups, the mean degree—the average number of edges incident on a node belonging to the network—was highest at scale 3 for a wide range of correlation thresholds (0.01<*R*<0.8). The mean characteristic path length (λ) for both groups, when controlled for the degree of the network (1<λ<1.57), showed similar trends at all three scales. The clustering coefficient (γ) for both groups, when controlled for the degree of the network, was highest at scale 3. Due to higher mean γ values, the small-world measure σ (γ/λ), when controlled for degree of the network, was highest at scale 3 for both groups. The small-world property (σ>1) showed a monotonic increase in small-worldness as the threshold increased and the degree decreased. σ values for higher correlation thresholds are difficult to interpret because at higher threshold values, graphs of functional brain networks have fewer edges (smaller degree) and tend to split into isolated subgraphs. Graph metrics such as clustering coefficient, characteristic path length, and small-world property do not meaningfully characterize network structures that are not composed of a single, large group of interconnected nodes [30].

Since functional connectivity and small-world properties were highest (*p*<0.01, Kolmogorov-Smirnov test) at lower-frequencies (scale 3: 0.01– 0.05 Hz) for both children and young-adults, we focus on this frequency interval in subsequent analyses, consistent with other recent studies [34],[35].

### Comparison of Small-World Metrics in Children and Young-Adults

We examined path length (λ), clustering coefficient (γ), and small-worldness (σ) values in the two groups in scale 3 (0.01–0.05 Hz). For group comparisons, we controlled for the average correlation value (*r*), as it varies considerably across individuals. Thus, for a given correlation threshold, the number of edges in the graph are likely to be different, resulting in different λ and γ values. To ensure that graphs in both groups had the same number of edges, individual correlation matrices were thresholded such that the resultant graph had on average K′ edges per node. K′ is the average number of edges per node in the graph obtained by thresholding individual correlation matrices with *R* = *r*_i_ (*r*_i_ is the average correlation value for subject *i*, *i* = 1–45), averaged across subjects. This procedure not only ensured that both groups had the same number of edges, but also selected a conservative K′ such that the networks generated were not disconnected. This is particularly important for network characterization because graph metrics are not interpretable when the network is disconnected. The value of K′ selected according to this procedure was 48 for both the groups. Thus, every network generated by using this degree preserving threshold will have exactly 2,160 ( = 48×90/2) edges, which is equivalent to a network cost of 0.54 ( = 2,160/4,005). Network cost is defined and calculated as the ratio of number of edges in the network to the maximum possible edges in the network [50]. Mean λ, mean γ, and mean σ values for the networks of each group were derived by thresholding the correlation matrices such that the network has on average K′ ( = 48) edges per node. Using this approach, no significant differences in the mean λ, γ, and σ values were observed between children and young-adults.

### Comparison of Global Efficiency of Whole-Brain Functional Connectivity in Children and Young-Adults

Global efficiency, the harmonic mean of the minimum path length between each pair of nodes, is an alternative measure of connectivity of the network. This measure overcomes some of the limitations of the original measure of network connectivity, characteristic path length, which is susceptible to disconnected nodes. We examined global efficiency (*E*_global_) values obtained for the functional brain networks constructed by thresholding (threshold values ranged from 0.01 to 0.99 with an increment of 0.01), the wavelet correlation matrix at each of the three scales. The mean *E*_global_ for both groups, when controlled for the degree of the network, was low (0.7<*E*_global_<1) and showed similar trends at all three scales. In the frequency interval 0.01–0.05 Hz (scale 3), mean *E*_global_ values for the two groups, obtained by thresholding the correlation matrices such that the network has on average K′ ( = 48) edges per node, which is equivalent to a network cost of 0.54 were compared. No significant differences in the mean *E*_global_ values were observed between the two groups.

### Classification Analysis of Whole-Brain Functional Connectivity in Children and Young-Adults

We examined differences in whole-brain functional connectivity patterns between children and young-adults. The connectivity patterns,- correlation values of 4,005 pairs of anatomical regions, were used as features in a support vector machine (SVM) classifier (see Text S1). We found that connectivity patterns in children could be distinguished from those in young-adults with accuracies ranging from 89% to 91%, with the highest accuracy in scale 3 (see Table 2). This suggests that functional connectivity patterns at the whole-brain level in children are significantly different from those in young-adults. We report below the nature of these developmental changes in the context of hierarchical and regional organization of brain connectivity.

**Table 2:** Whole-brain functional connectivity patterns in children and young-adults are significantly different.

Scale	Frequency Range (Hz)	Accuracy Percent
1	0.13–0.25	88.89
2	0.06–0.12	88.89
3	0.01–0.05	91.11

A SVM based classifier was used to examine differences in whole-brain functional connectivity patterns between children and young-adults. Connectivity patterns were classified with high accuracy and accuracy was highest in scale 3, which corresponds to low-frequency fluctuations (0.01–0.05 Hz). Scale 1: 0.13–0.25 Hz and scale 2: 0.06–0.12 Hz.

### Comparison of Hierarchical Organization of Whole-Brain Functional Connectivity in Children and Young-Adults

**Figure 2:**
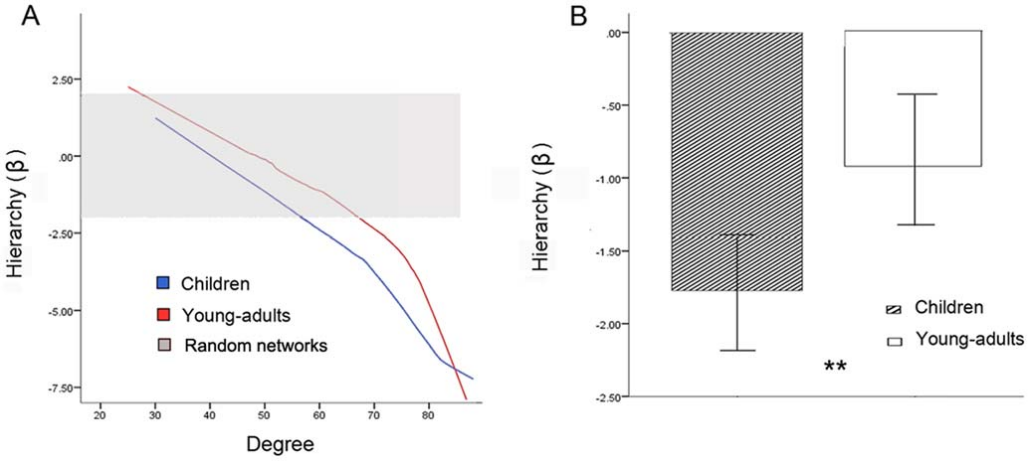
Developmental changes in hierarchical organization of whole-brain functional connectivity network. (A) Hierarchy measure (β), for children (blue) and young-adults (red) at scale 3 (0.01– 0.05 Hz). The β values for both groups are high (β z-scores ranged from −7.5 to 2.5), and are significantly greater than the β values obtained from random networks (β_random_ z-scores ranged from −1.96 to 1.96, indicated in gray). (B) Mean β values were significantly higher in young-adults (indicated by **) compared to children (*p*<0.01). Error bars represent standard error.

Hierarchy (β) is a measure of the relationship between the clustering coefficient and number of nodes in the network. Networks with higher hierarchy values are characterized by high degree nodes, which exhibit low clustering, and vice versa. These hierarchical networks contain small densely connected clusters; these clusters combine to form large less-interconnected clusters, which combine again to form larger lesser-interconnected clusters [51]. We examined β values obtained for the functional brain networks constructed by thresholding (threshold values ranged from 0.01 to 0.99 with an increment of 0.01) the wavelet correlation matrix at scale 3 (0.01–0.05 Hz). As shown in Figure 2A, the β values for both groups, when controlled for the degree of the network, were significantly higher (−7.5<β<2.5) than β values obtained from random networks (*p*<0.01). Furthermore, β values in the young-adult group were significantly higher than in the child group (*p*<0.001, Kolmogorov-Smirnov Test). The mean β value for the two groups, obtained by thresholding the correlation matrices such that the network has on average K′ ( = 48) edges per node, which is equivalent to a network cost of 0.54, was significantly higher in young-adults than in children (*p*<0.01), as shown in Figure 2B.

### Comparison of Regional Differences in Network Organization and Connectivity in Children and Young-Adults

**Figure 3:**
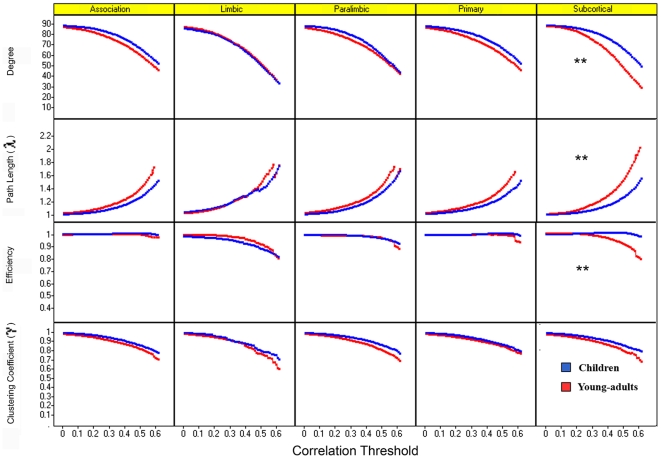
Developmental changes in network metrics for five major functional divisions of the human brain. Graph metrics—degree, path length (λ), efficiency, clustering coefficient (γ), within each of the five divisions: association, limbic, paralimbic, primary, and subcortical—are shown for children (blue) and young-adults (red), as a function of the correlation threshold. In the subcortical division, for threshold values from 0.1 to 0.6, degree and efficiency values were significantly higher and λ values significantly lower in children, compared to young-adults (*p*<0.01, indicated by **), while for the association, limbic, paralimbic, and primary sensory areas, no significant differences in the degree, λ, efficiency, and γ values were observed at any correlation threshold.

We then examined regional differences in network organization of five major divisions—association, limbic, paralimbic, primary, and subcortical areas [45]—with the rest of the brain. Figure 3 shows a plot of degree, path length (λ), efficiency, and clustering coefficient (γ) values for each of the five areas, for children and young-adults, as a function of the correlation threshold. In the subcortical division, the fitted growth curve of degree and efficiency values was significantly higher (*p*<0.01) while the curve of λ values was significantly lower (*p*<0.01) in children, compared to young-adults, reflecting higher connectivity, higher efficiency values, and lower path length for a range of threshold values from 0.1 to 0.6. A similar analysis in the association, limbic, paralimbic, and primary sensory areas, revealed no significant differences in the degree, λ, efficiency, and γ values. Across the five divisions, no significant differences in the degree, λ, efficiency, and γ values were observed for correlation threshold values >0.6, mainly due to the large variance observed at higher threshold values.

We next examined the degree, λ, efficiency, and γ values for each of the 90 anatomical ROIs, for the two groups. Consistent with the above findings, a significant number of subcortical areas (six out of eight; *p*<0.01) showed differences between the two groups in at least one of the four metrics (degree, λ, efficiency, and γ), whereas only two out of eight regions in the primary sensory, 17 out of 44 regions in association, three out of six regions in limbic, and 12 out of 24 regions in the paralimbic areas, showed differences (see Table S1 for regions that showed significant differences in degree, λ, efficiency, and γ values between the two groups).

We next examined connectivity differences within each of the five functional subdivisions. Connectivity differences here reflect the change in the strength of interregional correlations in spontaneous blood oxygen level-dependent fluctuations. The functional connectivity within the paralimbic areas was significantly higher in the young-adults, compared to children (*p*<0.001; *p*<0.01, false discovery rate (FDR)-corrected for multiple comparisons). There were no differences in functional connectivity within the association, limbic, primary, and subcortical areas.

### Interregional Functional Connectivity Changes with Development

**Figure 4:**
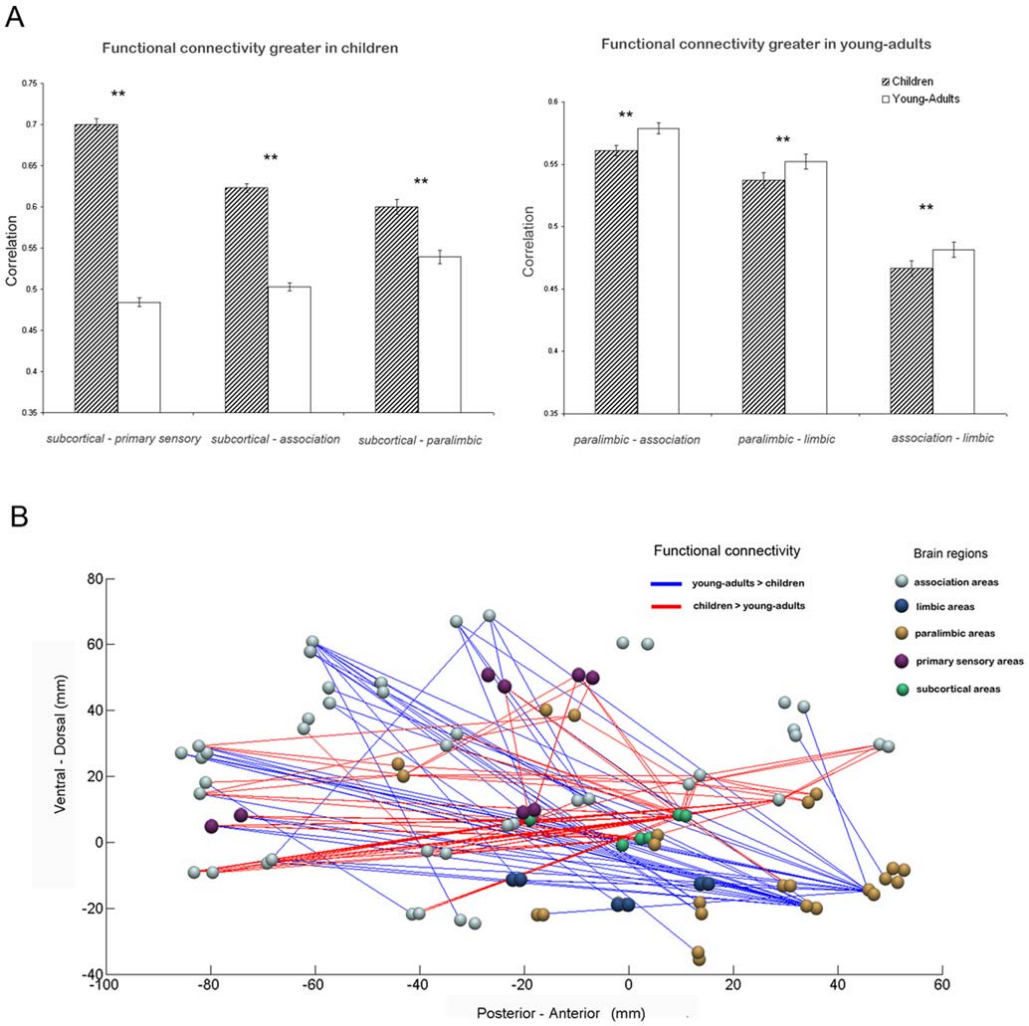
Developmental changes in interregional functional connectivity. (A) Children had significantly greater subcortical-primary sensory, subcortical-association, subcortical-paralimbic, and lower paralimbic-association, paralimbic-limbic, association-limbic connectivity than young-adults (*p*<0.01, indicated by **). Error bars represent standard error. (B) Graphical representation of developmental changes in functional connectivity along the posterior-anterior and ventral-dorsal axes, highlighting higher subcortical connectivity (subcortical nodes are shown in green) and lower paralimbic connectivity (paralimbic nodes are shown in gold) in children, compared to young-adults. Brain regions are plotted using the *y* and *z* coordinates of their centroids (in mm) in the MNI space. 430 pairs of anatomical regions showed significantly higher correlations in children and 321 pairs showed significantly higher correlations in young-adults (*p*<0.005, FDR corrected). For illustration purposes, the plot shows differential connectivity that were most significant, 105 pairs higher in children (indicated in red) and 53 higher in young-adults (indicated in blue), (*p*<0.0001, FDR corrected).

To further investigate regional differences in network organization, we examined interregional connectivity differences between the two groups. We found that the subcortical areas had increased connectivity with the primary sensory, association, and paralimbic areas in children, compared to young-adults. Young-adults, on the other hand, had increased connectivity between paralimbic and association areas, between paralimbic and limbic areas, and between limbic and association areas (*p*<0.001; *p*<0.01, FDR-corrected for multiple comparisons) (Figure 4A). The classification analysis of interregional connectivity showed complementary set of findings (see Text S1 for details). The interregional connectivity patterns in children could be distinguished from those in young-adults with accuracies ranging from 44% to 91%, with high accuracy values observed for connectivity patterns between subcortical areas and the primary sensory (91%), association (90%) and paralimbic (83%) areas, and between paralimbic and association (80%) areas (see Table 3). Figure 4B shows a graphical representation of developmental differences in functional connectivity along the posterior-anterior and ventral-dorsal axes, highlighting greater subcortical connectivity in children and greater paralimbic connectivity in young-adults. Figure S1 shows separate group-averaged functional connectivity matrices for children and young-adults, and Text S1 provides information about interparticipant variability in these matrices.

**Table 3:** Interregional functional connectivity patterns in children and young-adults are significantly different.

Interregional Functional Connectivity	Accuracy Percent
Subcortical – Primary sensory	91
Subcortical - Association	90
Subcortical – Paralimbic	83
Paralimbic – Association	80
Paralimbic – Primary sensory	78
Association – Primary sensory	78
Association – Limbic	78
Limbic – Paralimbic	76
Limbic – Subcortical	64
Limbic – Primary sensory	44

A SVM classifier was used to examine differences in interregional functional connectivity patterns between children and young-adults in scale 3. Pairs of regions are rank-ordered by classification rates. Classification accuracy ranged from 44% to 91%.

### Developmental Changes in Functional Connectivity with Wiring Distance

**Figure 5:**
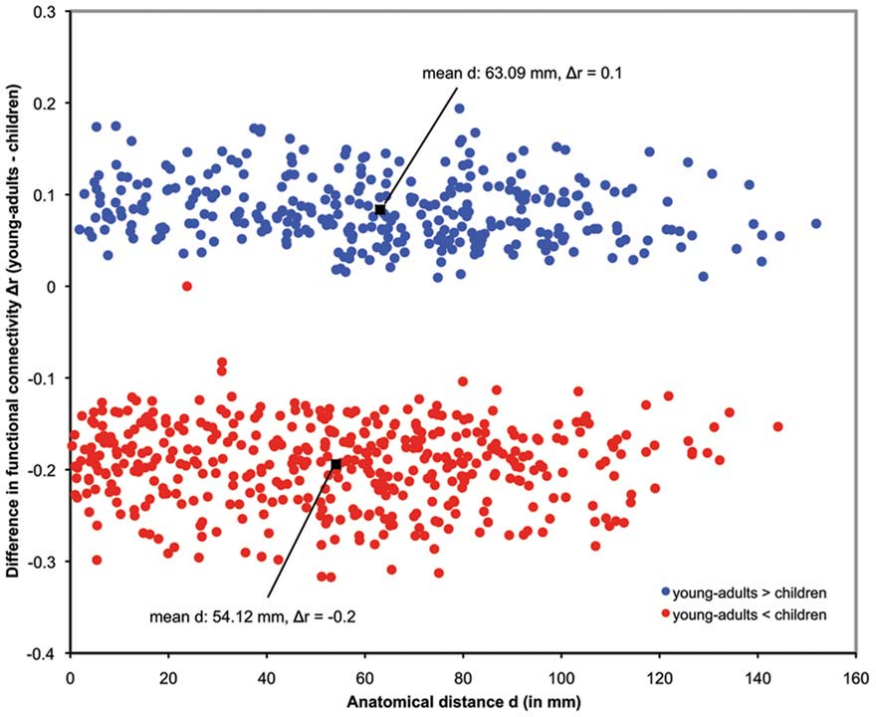
Developmental changes in functional connectivity with DTI-based wiring distance. The wiring distance (d) of all connections which differed significantly between the children and young-adults is plotted against developmental change in functional correlation values (Δ*r*) of those connections. Correlation values that were higher in children, compared to young-adults, are displayed in red, and the values that were higher in young-adults, compared to children, are displayed in blue. The mean wiring distance of the connections that showed higher correlation values in children (mean Δ*r* = −0.2), compared to young-adults, was 54.12 mm; the mean wiring distance of the connections that showed higher correlation values in young-adults (mean Δ*r* = 0.1), compared to children, was 63.09 mm. The correlation values of short-range connections were significantly greater in children whereas young-adults showed stronger long-range connectivity (*p*<0.0001). Wiring distances were computed using DTI-based fiber tracking.

Lastly, we investigated whether development is associated with simultaneous emergence of functional segregation and integration at the whole-brain level. For each pair of ROIs we first computed the wiring distance using DTI-based fiber tracking. We computed the fiber length in a common Montreal Neurological Institute (MNI) space rather than individual subject space to rule out any potential confounding effects of developmental changes in interregional fiber length on our findings. We then examined developmental changes in functional connectivity in relation to the wiring distance between them. We found that functional connectivity between more proximal anatomical regions were significantly higher in children, whereas functional connectivity between more distal anatomical regions were significantly higher in young-adults (*p*<0.0001), as shown in Figure 5. This suggests a pattern of higher short-range functional segregation in children and higher long-range functional integration in young-adults.

To further examine the robustness of our findings, we repeated our functional connectivity versus wiring distance analysis using Euclidean distance instead of DTI-based wiring distance. The results were highly consistent with those reported above: functional connectivity between more proximal anatomical regions in Euclidean space was significantly higher in children, whereas functional connectivity between more distal anatomical regions in Euclidean space was significantly higher in young-adults (*p*<0.0001).

## Discussion

To our knowledge, this is the first study to characterize the organization and development of large-scale human brain networks in children. We used graph-theoretical metrics to measure and characterize global and local functional brain organization in children and young-adults. The main findings of our study are: (1) large-scale brain networks in 7–9-y-old children showed similar small-world, nonrandom, functional organization at the global level, as young-adults; (2) compared to young-adults, functional brain networks in children showed significantly lower levels of hierarchical organization; (3) children and young-adults had significantly different interregional connectivity patterns, more specifically stronger subcortical-cortical and weaker cortico-cortical connectivity in children; and (4) the development of large-scale brain connectivity involves functional segregation and integration, characterized by a shift from stronger short-range connections in children to stronger long-range connections in young-adults. Collectively, these and other findings reported here provide new insights into the development of large-scale brain organization in children.

### Small-World Functional Organization in Children

A small-world network is characterized by a high clustering coefficient and a low characteristic path length. Functional brain networks in both children and young-adults showed small-world properties (σ_children_>1, σ_young-adults_>1) suggesting the presence of subnetworks of densely connected nodes, mostly connected by a short path. Similar findings were observed when clustering coefficient and global efficiency were used as alternative measures of small-worldness.

Small-world characterization is well-suited for analyzing functional brain networks at the systems level because these networks are complex and optimally connected to minimize information processing costs [36],[52]. Functional connectivity networks of the human brain constructed from EEG as well as MEG data have also been shown to have small-world architecture [38],[39]. Salvador et al. [53] examined connectivity in task-free functional MRI data with the same 90 ROI parcellation scheme used in our study and they reported small-world architecture in this network. This finding was replicated by Achard et al., who also reported that small-world properties were salient in the low frequency interval 0.03–0.06 Hz [35] in adults (ages 25–35 y), and by Supekar et al. in older adults (ages 37–77 y) [34]. These findings, primarily derived from functional data obtained from middle-age to older adults, suggest that the functional organization of the brain has a small-world architecture, a characteristic that may assist in robust and dynamic information processing.

Our finding that large-scale brain networks in children showed small-world properties that were very similar to young-adults, together with the above observations, suggests that key aspects of functional brain organization are conserved throughout the developmental process—from early childhood to young adulthood and into older adulthood. Critically, despite the fact that the brain undergoes vast structural reorganization at the neuronal level in the form of myelination and synaptic pruning throughout development, key global properties of functional organization appear to be conserved.

### Functional Brain Connectivity Patterns in Children and Young-Adults Are Significantly Different

Notwithstanding similarities in global, whole-brain, small-world network properties, functional connectivity patterns in children were significantly different from those in young-adults. SVM-based pattern classification analysis showed that connectivity patterns in children could be distinguished from those in young-adults with an accuracy of over 90%. Accuracy was highest (91%) for connectivity patterns in the low frequency interval (scale 3; 0.01–0.05 Hz). Previous studies have reported that resting-state functional connectivity is most robust at frequencies below 0.1 Hz [20],[54],[55] and that these low frequency fMRI fluctuations are related to interregional coupling of local field potentials in the gamma band [56],[57]. Overall, these findings suggest that observed developmental changes in the functional connectivity measured by fMRI resting state signals are likely to reflect underlying differences in coupling of neuronal signals. We discuss below the nature of developmental changes in the context of hierarchical and regional organization of brain connectivity.

### Development of Functional Hierarchical Organization

Our data provide new evidence that large-scale brain networks in children and young-adults differ in their hierarchical organization. Children showed significantly lower (*p*<0.001) levels of hierarchical organization than young-adults. Hierarchical networks are characterized by the presence of small densely connected clusters; these clusters combine to form large less-interconnected clusters, which combine again to form larger lesser-interconnected clusters [51]. Hierarchical organization has been discovered in the World Wide Web and several biological networks [40],[58],[59]. In a recent study, Bassett and colleagues reported significant levels of hierarchical organization in anatomical human brain networks based on interregional correlations in cortical thickness [40]. Our study extends these findings to the realm of hierarchical organization in functional human brain networks in not only young-adults but also in children. Hierarchical networks are optimally connected to support top-down relationships between nodes and minimize wiring costs, but are vulnerable to attack on hubs [51]. The presence of hierarchical organization in the large-scale brain networks of children and young-adults suggests efficient functional connectivity patterns within these networks at the expense of higher vulnerability to attacks. Lower levels of hierarchical organization in children may therefore be protective to such vulnerability, allowing for more flexibility in network reconfiguration on the basis of individual differences in cognitive experience and reserve. How modularity and hierarchy emerge in functionally meaningful ways is an important topic for future research, but the important finding here is that quantitative measures of hierarchy can be used to examine the emergence of functional hierarchy in the developing brain.

### Development of Interregional Functional Connectivity

We used the parcellation scheme of Mesulam to examine developmental changes in the functional connectivity of five major functional divisions of the human brain. Briefly, the primary sensory division consists of unimodal regions for processing visual, auditory, somatosensory, olfactory, and gustatory signals. The subcortical division includes deep brain nuclei, notably the basal ganglia and thalamus, and the association division comprises higher order multimodal regions, including the lateral prefrontal, parietal, and temporal cortices. The paralimbic division consists of the insula, anterior cingulate cortex, posterior cingulate cortex and the orbitofrontal cortex, and the limbic division includes the amygdala and hippocampus. Together, these divisions map the external world into brains' internal sensory, attentional, mnemonic, emotional, and motivational systems [60].

Graph-theoretical analysis identified subcortical regions as a major locus of between-group differences in brain connectivity. More specifically, subcortical connectivity was characterized by higher degree, lower path length and higher efficiency in children (Figure 3). Node wise analysis showed that the caudate, putamen, and thalamus all showed higher degree, lower path length, and higher efficiency in children. The globus pallidus was the only subcortical region that did not differ in these network metrics between children and young-adults. Further analysis of functional connectivity with the other four subdivisions revealed that subcortical areas were more strongly correlated with primary sensory, association, and paralimbic areas in children, as shown in Figure 4A. These results suggest that subcortical-cortical connections are both more profuse and stronger in children and that the functional development of subcortical connectivity is characterized by both changes in wiring and strength of connections.

We also detected significant differences in cortical connectivity but in this case the pattern of age-related differences was reversed, with children showing significantly weaker connectivity between paralimbic, association, and limbic areas (Figure 4A). Graph-theoretical measures of degree, efficiency, and path length of the four cortical subdivisions did not differ between the two groups (Figure 3). This suggests that key aspects of cortico-cortical wiring are similar in children and young-adults but the strength of the connections is weaker in children.

These developmental changes converge on and extend findings from structural neuroimaging studies that have shown protracted age-related structural differences in the regional gray- and white-matter [6]–[9],[15]. Our findings of differences in subcortical connectivity is consistent with reports that these areas undergo massive structural rewiring characterized by progressive myelination of axons that emanate from these regions followed by extension of these myelinated axons into the cortex during development [11],[15]. The later teen years, which span an interval in between childhood and young-adulthood is a period of significant brain maturation [61]. In particular, caudate, putamen, and thalamus regions of the subcortical division show some of the largest changes in fractional anisotropy of white-matter tracts, increasing almost 30% to 50% from 5 to 25 years of age. In contrast, major cortico-cortico tracts show a more modest increase of 8% to 20% [15]. Taken together, these results suggest that changes in interregional functional connectivity parallel changes in maturation of white-matter tracts between childhood and young-adulthood. Critically, our data provide novel evidence for a process of rewiring and pruning of subcortical-cortical connectivity accompanied by increased cortico-cortical connectivity at the functional level.

Subcortical areas, comprising the basal ganglia and the thalamus, are important for adaptive processing of distributed information in a manner that facilitates the transformation of sensory input and cognitive operations into behavior [62]. More specifically, the basal ganglia link signals in distinct functional networks during different phases of cognitive information processing [63]. Neurophysiological models and anatomical tracing studies have provided evidence for parallel motor, limbic, and prefrontal cortico-basal-ganglia loops [64],[65], which funnel large-scale cortical activity into behaviorally relevant motor output. In humans, these circuits are characterized by segregated and overlapping connectivity patterns and a complex pattern of hierarchically organized frontal inputs [66],[67]. These patterns support the parallel flow of cortical signals inputs into the basal ganglia, where multiple reward related signals are integrated in ways that facilitate incentive learning over short time scales and habit formation over long time scales [68],[69]. There have been few studies of how these loops develop in children, but the pattern of changes in subcortical-cortical functional connectivity observed in our study suggest a process of pruning at the systems-level. This form of pruning is characterized by weakening of specific subcortical links, leading to longer path lengths similar to those seen in young-adults. Exactly how these links result in the formation of parallel and integrative loops, which support large-scale neuronal networks for learning and memory [63],[70] remains to be investigated.

Changes in paralimbic connectivity were the cornerstone of developing cortico-cortico connectivity. Paralimbic areas play a major role in detection of salient environmental events [71], in facilitating flexible behaviors in response to risk, reward, and punishment [72],[73], and in goal directed behavior [74]. Converging evidence from a number of brain imaging studies across several task domains suggests that the insula and the anterior cingulate cortex respond to the degree of subjective salience, whether cognitive, homeostatic, or emotional [75],[76]. These paralimbic areas play a causal role in activating attentional and memory systems within association areas to facilitate controlled processing of stimuli during cognitively demanding tasks [71]. Paralimbic and association areas also moderate emotional reactivity to stimuli in limbic areas [77],[78]. These core motivational and regulatory processes are known to undergo significant changes during adolescence [79], a time when coordinated interaction of emotion, reasoning, and decision-making becomes increasingly important [80],[81]. The tighter integration of paralimbic with association and limbic areas revealed by our study may underlie the large-scale functional changes that facilitate this critical developmental process.

### Short- and Long-Range Functional Connectivity in Children Compared to Young-Adults

Our analysis of functional connectivity changes with wiring distance provides strong evidence that development is characterized by simultaneous reduction of short-range connectivity and strengthening of long-range connectivity. This suggests a process of greater functional segregation in children and greater functional integration in young-adults at the whole-brain level, not just in circumscribed nodes of the attentional control [27] and default node networks [28]. In contrast to the 90 cortical and subcortical nodes, based on whole-brain parcellation [82], used in our study, Fair and colleagues [27],[28] focused their analysis on 39 distinct cortical regions involved in task-control and default-mode networks. Whereas the lack of correspondence between specific brain regions in the two studies makes a detailed comparison difficult, our findings are, nevertheless, consistent with distributed changes in these two large-scale networks reported by Fair and colleagues. Methodologically, our studies are an improvement over prior studies because we used continuous resting state fMRI data, rather than resting state data extracted from intertask rest periods, uncontaminated by cognitive tasks. Furthermore, our findings indicate that simultaneous weakening of short-range connections and strengthening of long-range connections changes with actual anatomical (physical) distance, derived from DTI data, rather than the Euclidean distance, between nodes. Our findings provide new and more direct evidence that dual changes in functional integration and segregation with wiring distance reflects a general developmental principle that operates at the level of the whole brain.

Two neurobiological processes are likely to contribute directly to these observed effects. One, systematic pruning of local connections with age are likely to result in weakening of local connections and formation of more localized and specialized processing nodes. These changes are known to occur prenatally, in childhood and in adolescence [83]. In parallel, increased myelination of axonal fiber tracks with age, also contribute to strengthening of long-range connectivity [84]. Both these processes are likely to be influenced by experience dependent Hebbian plasticity, leading to selective strengthening and weakening of connections [85].

This selective strengthening and weakening of connections may be additionally influenced by developmental changes in interregional wiring distance. For example, on the basis of previous findings of an inverse relationship between strength of functional connectivity and wiring distance in adults [53],[86], the observed age-related decrease in subcortical-cortical functional connectivity may be due to age-related increases in subcortical-cortical wiring distance. In our analysis, however, we controlled for any confounding influences of changes in physical wiring distance by computing functional connectivity and wiring distance in a common MNI space. Further studies that examine both functional connectivity and wiring distance in native image space are needed in order to investigate the influence of age-related changes in wiring distance on the observed age-related changes in functional connectivity. More generally, the manner in which these structural and functional changes in connectivity influence the development of large-scale functional organization in the human brain is an important topic for future research. Recent studies do, however, suggest that intrinsic resting-state functional connectivity in the human brain reflects anatomical connectivity at both short and long spatial scales [87],[88]. Taken together, these findings suggest that the development of large-scale functional connectivity is related to ongoing developmental changes in structural connectivity.

### Conclusion

Our findings suggest that large-scale brain networks derived from task-free fMRI have a robust functional organization in 7–9-y-old children. Importantly, we show that the dynamic process of over-connectivity followed by pruning, which rewires connectivity at the neuronal level [89], also operates at the systems level and helps reconfigure and rebalance subcortical and paralimbic connectivity in the developing brain. Our study demonstrates the usefulness of network analysis of functional connectivity in elucidating the principles underlying brain maturation. Furthermore, our study shows how quantitative analysis of anatomical connectivity, and in particular the computation of wiring distance between brain regions, allows us to link changes in functional networks to the maturation of white matter tracts. Such multimodal analysis of structural and functional brain connectivity will prove useful in helping us better understand the network architecture that shapes and constrains cognitive development.

More generally, our findings provide a framework for examining how fundamental aspects of large-scale organization are disrupted in neurodevelopmental disorders. Previous work has suggested that resting-state functional connectivity can be used to assess disrupted connectivity between specific brain regions that are relevant to the disease-specific pathology in neurodevelopmental disorders such as autism spectrum disorders [90], and attention-deficit/hyperactivity disorder [91], disorders that are thought to be characterized by disruptions in synaptic pruning and myelination at the neuronal level [92]–[94]. The methods and results developed here provide a template for a more detailed investigation of disruptions in the large-scale organization of brain networks in these and other developmental brain disorders.

## Materials and Methods

### Participants

Twenty-three children and 22 IQ-matched young-adult subjects participated in this study after giving written, informed consent. For those subjects who were unable to give informed consent, written, informed consent was obtained from their legal guardian. The study protocol was approved by the Stanford University Institutional Review Board. The children subjects (10 males, 13 females) ranged in age from 7 to 9 y (mean age 7.95 y) with an IQ range of 88 to 137 (mean IQ: 112); the young-adult subjects (11 males, 11 females) ranged in age from 19 to 22 y (mean age 20.4 y) with an IQ range of 97 to 137 (mean IQ: 112). The subjects were recruited locally—children from local schools and young-adults from Stanford University and neighboring community colleges. Eleven of 23 children subjects were 2nd graders and the rest of the children subjects were 3rd graders; the young-adult subjects had 13 to 16 y of education (mean years of education 14.5).

### Data Acquisition

For the task-free scan, subjects were instructed to keep their eyes closed and try not to move for the duration of the 8-min scan. Functional Images were acquired on a 3T GE Signa scanner (General Electric) using a custom-built head coil. Head movement was minimized during scanning by a comfortable custom-built restraint. A total of 29 axial slices (4.0 mm thickness, 0.5 mm skip) parallel to the AC-PC line and covering the whole brain were imaged with a temporal resolution of 2 s using a T2* weighted gradient echo spiral in-out pulse sequence [95] with the following parameters: TR = 2,000 msec, TE = 30 msec, flip angle = 80 degrees, 1 interleave. The field of view was 20 cm, and the matrix size was 64×64, providing an in-plane spatial resolution of 3.125 mm. To reduce blurring and signal loss arising from field inhomogeneities, an automated high-order shimming method based on spiral acquisitions was used before acquiring functional MRI scans. A high resolution T1-weighted spoiled grass gradient recalled (SPGR) inversion recovery 3D MRI sequence was acquired to facilitate anatomical localization of functional data. The following parameters were used: TI = 300 msec, TR = 8.4 msec; TE = 1.8 msec; flip angle = 15 degrees; 22 cm field of view; 132 slices in coronal plane; 256×192 matrix; 2 NEX, acquired resolution = 1.5×0.9×1.1 mm. Structural and functional images were acquired in the same scan session.

### Data Preprocessing

Data were preprocessed using statistical parametric mapping (SPM5) software (http://fil.ion.ucl.ac.uk/spm). The first eight image acquisitions of the task-free functional time series were discarded to allow for stabilization of the MR signal. Each of the remaining 232 volumes underwent the following preprocessing steps: realignment, normalization to the MNI template, and smoothing carried out using a 4-mm full-width half maximum Gaussian kernel to decrease spatial noise. Excessive motion, defined as greater than 3.5 mm of translation or 3.5 degrees of rotation in any plane, was not present in any of the task-free scans.

### Anatomical Parcellation

The preprocessed task-free functional MRI datasets were parcellated into 90 cortical and subcortical regions using anatomical templates defined by Tzourio-Mazoyer et al. [82]. A task-free fMRI timeseries was computed for each of the 90 regions by averaging all voxels within each region at each time point in the time series, resulting in 232 time points for each of the 90 anatomical regions of interest. These regional fMRI time series were then used to construct a 90 node whole-brain task-free functional connectivity network for each subject.

### Construction of Large-Scale Whole-Brain Functional Connectivity Network

Wavelet analysis was used to construct correlation matrices from the regional fMRI time series data. These matrices described frequency-dependent correlations, a measure of functional connectivity, between spatially distinct brain regions. Correlation matrices were then thresholded to generate a whole-brain functional connectivity network.

Wavelets are mathematical functions that transform the input signal into different frequency components [96]. Wavelets methods have previously been applied in the analysis of task-based as well as task-free fMRI signal [35],[97]. In our study, we applied a maximum overlap discrete wavelet transform (MODWT) to each of the 90 regional time series from each subject to obtain the contributing signal in the following three frequency components: scale 1 (0.13–0.25 Hz), scale 2 (0.06–0.12 Hz), and scale 3 (0.01–0.05 Hz). To account for a relatively small number (232) of data points per time series for low frequency correlation analysis, the vector representing the time series beyond its boundaries (<0 and >232) was assumed to be a symmetric reflection of itself. At each of the three scales, wavelet correlations between signals in the 90 anatomical regions were determined by computing the correlation coefficient between the transformed signals at that scale.

For each subject, a 90-node, scale-specific, undirected graph of the functional connectivity network was constructed by thresholding the wavelet correlation matrix computed at that scale. If the wavelet correlation value between two anatomical regions represented by nodes *i* and *j* in the network exceeded a threshold, then an edge was drawn between node *i* and node *j*. There is currently no formal consensus regarding threshold selection, so we computed networks for threshold values from 0.01 to 0.99 with an increment of 0.01. Once a whole-brain functional connectivity network was constructed from the correlation matrix, we characterized this network using graph theoretic metrics of global and local brain organization including small-worldness and hierarchy.

### Small-World Analysis of the Whole-Brain Functional Connectivity

Small-world properties of a network are described by the clustering coefficient and the characteristic path length of the network. The clustering coefficient and characteristic path length of functional brain networks generated from the task-free fMRI data obtained from 23 children and 22 young-adults were computed. The clustering coefficient of every node was computed as the ratio of the number of connections between its neighbors divided by the maximum possible connections between its neighbors. The clustering coefficient (*C*) of the network was calculated as the mean of the clustering coefficients of all the nodes in the network. The mean minimum path length of a node was computed as the average of minimum distances from that node to all the remaining nodes in the network. The characteristic path length (*L*) of the network was the average of the mean minimum path lengths of all the nodes in the network. The clustering coefficient and path length of nodes completely disconnected with the network were set as 0 and Inf respectively, and these nodes were excluded while computing *C* and *L*. To evaluate the network for small-world properties, we compared the clustering coefficient and the characteristic path length of the network with corresponding values (*C*_ran_, *L*_ran_) obtained and averaged across 1,000 random networks with the same number of nodes and degree distribution [98]. The degree of every node (a measure of its connectivity) was calculated by counting the number of edges incident on that node. The mean degree of the network was the average of the degree of all the nodes in the network. Small-world networks are characterized by high normalized clustering coefficient γ (*C*/*C*_ran_)>1 and low normalized characteristic path length λ (*L*/*L*_ran_)≈1 compared to random networks [99]. A cumulative metric σ—the ratio of normalized clustering coefficient (γ) to the characteristic path length (λ), a measure of small-worldness—is thus greater than 1 for small world networks.

### Analysis of Global Efficiency of Whole-Brain Functional Connectivity

Small-world networks are characterized by high clustering coefficient and low characteristic path length. These small-world metrics, particularly the path length, are not meaningful when the graph contains disconnected nodes. To address this issue, we ensured that only small-world metrics computed on connected graphs were considered in our analysis. Specifically, the algorithm used to choose the correlation threshold (*R*) guaranteed that disconnected graphs were excluded from the analysis. Also, in the node-wise clustering coefficient comparison analysis, we only considered thresholds from 0.1 to 0.6. We chose these thresholds because beyond 0.6 the network gets divided into disconnected subset of nodes.

To determine if our characteristic path length findings were robust and reliable, we computed the efficiency of functional brain networks. It has been previously reported that efficiency as a graph metric (1) is not susceptible to disconnected nodes, (2) is applicable to unweighted as well as weighted graphs, and (3) is a more meaningful measure of parallel information processing than path length [50]. Efficiency of a graph (*E*_global−net_) [100] is the inverse of the harmonic mean of the minimum path length between each pair of nodes, *L*_ij_, and was computed as,
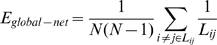
(1)To evaluate the network for its global efficiency of parallel information processing, we compared the global efficiency of the network (*E*_global−net_) with corresponding values (*E*_global−ran_) obtained and averaged across 1,000 random networks with the same number of nodes and degree distribution. A network with small-world properties is characterized by a global efficiency value that is lower than the random network: *E*_global_ (*E*_global−net_/*E*_global−ran_)<1.

### Analysis of Hierarchical Organization of Whole-Brain Functional Connectivity

We evaluated the hierarchical nature of the large-scale whole-brain functional connectivity network by the β parameter [51]. β measures the extent of the power-law relationship between the clustering coefficient (*C*) and the degree (*k*): *C*≈*k* ^−β^. The clustering coefficient (*C*_i_) and the degree (*k*_i_) of every node was computed; β of the network was calculated by fitting a linear regression line to the plot of log(*C*) versus log(*k*).

### Analysis of Regional Differences in Network Organization and Connectivity

The human brain can be divided into five major divisions—association, limbic, paralimbic, primary, and subcortical—each of them having a distinct function [45]. We assessed the network organization of these cortical divisions and how it differs in development by examining the regional profile of metrics (degree, λ, *E*_global_, and γ) at the divisional level. The 90 anatomical regions of our network were grouped into these five cortical divisions. The association division consists of 44 regions, the limbic division consists of 12 regions, the paralimbic division consists of 24 regions, the primary division consists of eight regions, and the subcortical division consists of eight regions (see Table S1 for region-wise division assignment). The graph metrics (degree, λ, *E*_global_, and γ) of 90 regions were aggregated into five divisions and the aggregated metrics in the two subject groups were compared using growth curve modeling, with an intercept, linear, and quadratic terms. In the aggregation step, the graph metric value at a correlation threshold of a division for a subject group was computed by averaging the corresponding metric values across regions belonging to that division. The aggregated metric values for threshold values from 0.1 to 0.6 were compared. We chose these thresholds because beyond 0.6 the network divides into disconnected subsets of nodes and small-world metrics are no longer meaningful [30]. This analysis was performed using the Mplus software (http://www.statmodel.com).

Growth curve models describe change (growth) with respect to a control variable. They are well-suited for analyzing group-level differences in biomedical data, particularly in cases where capturing and analyzing individual growth trajectories is important. Furthermore, for group comparisons, growth curve models alleviate the problem of multiple comparisons as fitted-curve coefficients are compared in contrast to traditional approaches where multiple individual points along the curve are compared. In our study, the growth trajectories of graph metric values of a subject carry important information about the variance within the group and needs to be incorporated in the model. The coefficients of growth curve models capture the baseline performance, instantaneous growth rate, and the acceleration of the variable of interest.

We next examined degree, λ, *E*_global_, and γ values for each of the 90 anatomical ROIs, for the two groups, as a function of the correlation threshold. The metric values for threshold values from 0.1 to 0.6 in the two subject groups were compared using growth curve modeling, as described above.

### Analysis of Interregional Functional Connectivity Changes with Development

To further characterize regional differences in network organization, we examined the regional connectivity at divisional level: association, limbic, paralimbic, primary, and subcortical. Differences in mean correlation coefficients for 4,005 pairs were aggregated into 15 pairs and the resulting differences were then normalized. (see also [101]). First, interregional pairs that showed statistically significant (*p*<0.01, FDR corrected) increased or decreased functional connectivity in young-adults group compared to child group were identified as (+1) or (−1), respectively. Second, the number of decreased (−1) or increased connectivities (+1) for each of the 15 pairs was counted. For example, to identify differential connectivity between the association division and the subcortical division, the number of decreased or increased connectivities between all pairs of subregions belonging to the association division and subcortical division was counted. Finally, since each brain region has a different number of subregions, the aggregated differential connectivity count was normalized by the number of possible connections between pairs of subregions belonging to the two divisions under investigation.

Next we examined regional correlation values (connectivity) in the two groups. We compared regional correlation values aggregated across the 4,005 pairs of anatomical regions, between young-adults and children. No significant between-group differences in the aggregated correlation values were observed. On the basis of this observation, subsequently, individual regional correlation values were z-transformed followed by centering of the distribution around zero mean. These normalized correlation values were compared between the two subject groups. t-Test with a false discovery rate of 0.005 was used to test for significant differences.

### Analysis of Developmental Changes in Functional Connectivity

We next examined the relationship between differences in regional correlation values (connectivity) in the two groups and the interregional wiring distance as determined using DTI. The wiring distance between two regions was computed by measuring the average length of the fiber tracks, in the MNI space, connecting those regions (see Text S1 for details).

## Supporting Information

Figure S1 **Functional connectivity in children and young-adults.** Group averaged functional connectivity matrices for children and young-adults. Value of the (*i,j*)th element of the connectivity matrix corresponds to group averaged scale 3 wavelet correlation between the resting-state timeseries of brain region *i* and region *j*. Low correlation values are shown in darker color whereas high correlation values are shown in lighter color. Qualitatively, children, compared to young-adults, showed higher connectivity between the subcortical (caudate, globus pallidus, putamen, thalamus) and the cortical regions, and lower connectivity between the paralimbic (cingulate gyrus, orbitofrontal cortex, insula, parahippocampus gyrus, rectus gyrus, temporal pole) and the cortical regions.
(2.70 MB TIF)

Table S1 **Graph metrics for each anatomical region.**
(0.17 MB DOC)

Text S1 **Experimental procedures.**
(0.07 MB DOC)

## Abbreviations

DTI: diffusion tensor imaging
FDR: false discovery rate
fMRI: functional magnetic resonance imaging
MNI: Montreal Neurological Institute
SVM: support vector machine
