# Supplementary figures and images for: Development of Large-Scale Functional Brain Networks in Children
Source: PLoS Biol. 2009 Jul 21;7(7):e1000157. doi: 10.1371/journal.pbio.1000157 (PMC2705656; doi:10.1371/journal.pbio.1000157)

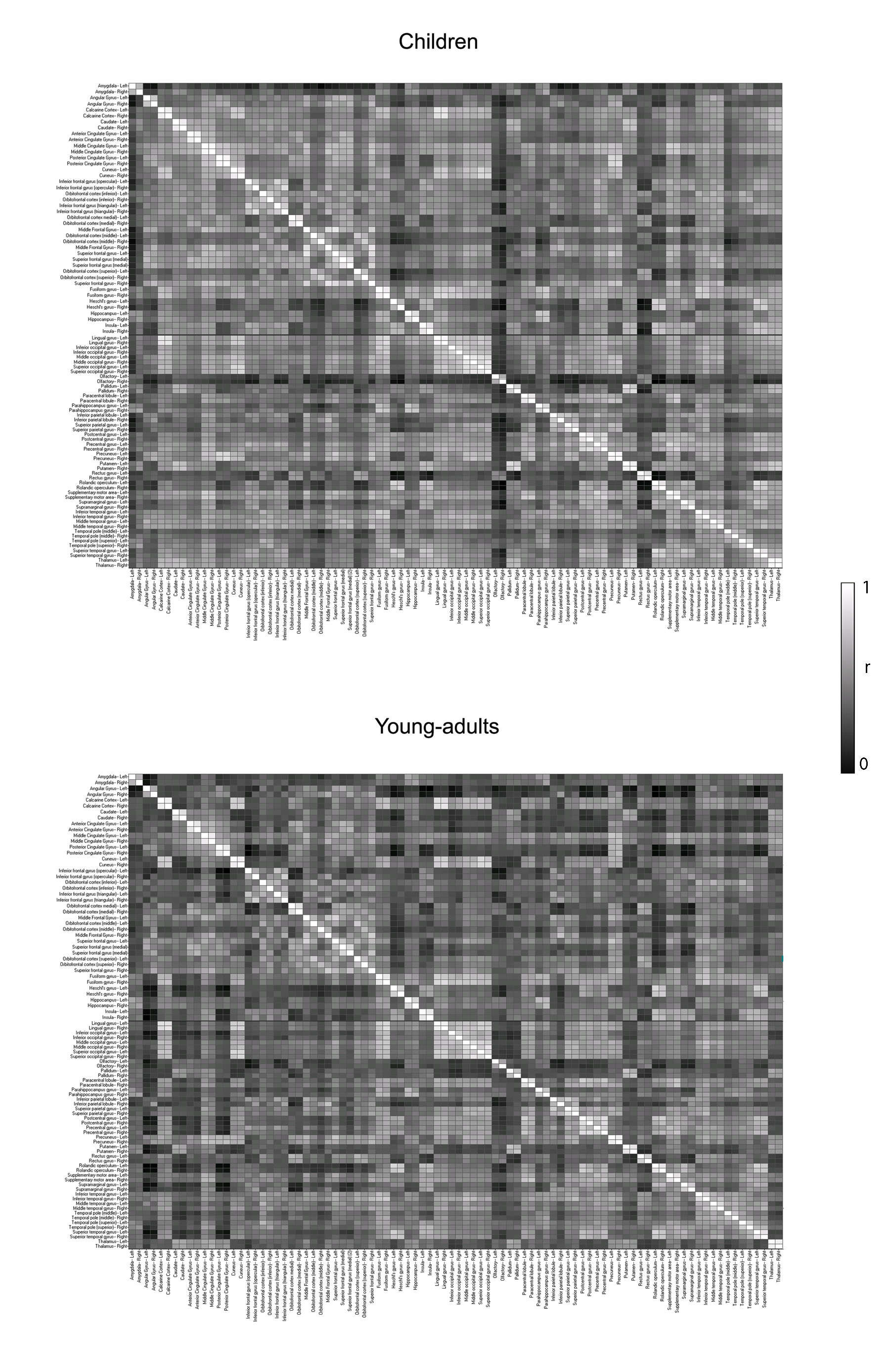

Supplement: Figure S1 — Functional connectivity in children and young-adults. Group averaged functional connectivity matrices for children and young-adults. Value of the (i,j)th element of the connectivity matrix corresponds to group averaged scale 3 wavelet correlation between the resting-state timeseries of brain region i and region j. Low correlation values are shown in darker color whereas high correlation values are shown in lighter color. Qualitatively, children, compared to young-adults, showed higher connectivity between the subcortical (caudate, globus pallidus, putamen, thalamus) and the cortical regions, and lower connectivity between the paralimbic (cingulate gyrus, orbitofrontal cortex, insula, parahippocampus gyrus, rectus gyrus, temporal pole) and the cortical regions. (2.70 MB TIF) [file pbio.1000157.s001.tif]
