# Supplementary material for: Development of Large-Scale Functional Brain Networks in Children
Source: PLoS Biol. 2009 Jul 21;7(7):e1000157. doi: 10.1371/journal.pbio.1000157 (PMC2705656; doi:10.1371/journal.pbio.1000157)
Supplement: Table S1 — Graph metrics for each anatomical region. (0.17 MB DOC) [file pbio.1000157.s002.doc]

**Supplementary Table S1. Graph metrics for each anatomical region.**

| | Brain Region | Class | **γ_i_**  Adult Children | | **λ_i_**  Adult Children | | Eglobal_i_  Adult Children | | K  Adult Children | | | --- | --- | --- | --- | --- | --- | --- | --- | --- | --- | | Amygdala, Left | Limbic | 1.28 | 1.02 | 1.19 | 1.29 | 0.85** | 0.68** | 30.64** | 18.91 | | Amygdala, Right | Limbic | 1.13 | 1.21 | 1.03 | 1.14 | 0.87 | 0.85 | 36.05 | 34.04 | | Angular Gyrus, Left | Association | 1.57 | 1.60 | 1.24** | 1.12** | 0.80** | 0.90** | 23.00** | 36.26** | | Angular Gyrus, Right | Association | 1.53 | 1.56 | 1.19** | 1.11** | 0.85** | 0.91** | 28.50** | 36.78** | | Calcarine Cortex, Left | Primary | 1.48 | 1.44 | 0.96 | 0.93 | 1.05 | 1.07 | 55.55 | 58.30 | | Calcarine Cortex, Right | Primary | 1.50 | 1.50 | 0.97 | 0.98 | 1.04 | 1.03 | 54.50 | 53.65 | | Caudate, Left | Subcortical | 1.33 | 1.43 | 1.17** | 0.97** | 0.88** | 1.03** | 33.68** | 52.91** | | Caudate, Right | Subcortical | 1.26 | 1.42 | 1.24** | 0.98** | 0.85** | 1.02** | 32.18** | 51.61** | | Anterior Cingulate Gyrus, Left | Paralimbic | 1.41 | 1.46 | 1.07** | 1.04** | 0.95** | 0.97** | 42.23** | 44.74** | | Anterior Cingulate Gyrus, Right | Paralimbic | 1.47 | 1.46 | 1.13** | 1.07** | 0.89** | 0.94** | 34.41** | 41.17** | | Middle Cingulate Gyrus, Left | Paralimbic | 1.27 | 1.32 | 0.85 | 0.84 | 1.14 | 1.15 | 67.73 | 69.13 | | Middle Cingulate Gyrus, Right | Paralimbic | 1.27 | 1.34 | 0.93 | 0.87 | 1.07 | 1.13 | 57.59 | 66.26 | | Posterior Cingulate Gyrus, Left | Paralimbic | 1.47 | 1.53 | 1.12** | 1.04** | 0.91** | 0.97** | 36.64** | 45.48** | | Posterior Cingulate Gyrus, Right | Paralimbic | 1.53 | 1.54 | 1.17** | 1.02** | 0.88** | 0.99** | 35.14** | 47.65** | | Cuneus, Left | Association | 1.56 | 1.52 | 1.02 | 1.00 | 0.99 | 1.01 | 47.77 | 50.30 | | Cuneus, Right | Association | 1.51 | 1.52 | 0.99 | 0.99 | 1.02 | 1.01 | 51.41 | 50.87 | | Inferior frontal gyrus (opercular), Left | Association | 1.56 | 1.57 | 1.16** | 1.02** | 0.88** | 0.99** | 33.32** | 48.00** | | Inferior frontal gyrus (opercular), Right | Association | 1.48 | 1.51 | 1.13** | 0.98** | 0.90** | 1.03** | 35.36** | 52.96** | | Orbitofrontal cortex (inferior), Left | Paralimbic | 1.37 | 1.36 | 0.96** | 0.99** | 1.05 | 1.02 | 55.05** | 51.09** | | Orbitofrontal cortex (inferior), Right | Paralimbic | 1.38 | 1.39 | 1.00** | 0.94** | 1.01 | 1.06 | 49.23** | 56.04** | | Inferior frontal gyrus (triangular), Left | Association | 1.41 | 1.53 | 1.08** | 0.98** | 0.95** | 1.02** | 42.32** | 52.00** | | Inferior frontal gyrus (triangular), Right | Association | 1.27 | 1.50 | 1.10** | 0.95** | 0.94** | 1.05** | 41.95** | 56.09** | | Orbitofrontal cortex medial), Left | Paralimbic | 1.48 | 1.48 | 1.05** | 1.11** | 0.96 | 0.91 | 43.41** | 37.57** | | Orbitofrontal cortex (medial), Right | Paralimbic | 1.44 | 1.43 | 1.04 | 1.12 | 0.97 | 0.92 | 43.91 | 39.26 | | Middle Frontal Gyrus, Left | Association | 1.35** | 1.46** | 0.96 | 0.97 | 1.04 | 1.04 | 54.09 | 54.26 | | Orbitofrontal cortex (middle), Left | Paralimbic | 1.48** | 1.50** | 1.07 | 1.23 | 0.95 | 0.83 | 41.59 | 27.61 | | Orbitofrontal cortex (middle), Right | Paralimbic | 1.52 | 1.49 | 1.16 | 1.23 | 0.86 | 0.83 | 30.14 | 28.22 | | Middle Frontal Gyrus, Right | Association | 1.33 | 1.45 | 1.01 | 0.96 | 1.00 | 1.05 | 48.18 | 55.22 | | Superior frontal gyrus, Left | Association | 1.35** | 1.47** | 0.91 | 0.96 | 1.09 | 1.05 | 60.36 | 55.17 | | Superior frontal gyrus (medial), Left | Association | 1.43** | 1.48** | 1.03** | 0.96** | 0.98 | 1.04 | 45.50** | 54.74** | | Superior frontal gyrus (medial), Right | Association | 1.45** | 1.47** | 1.06** | 0.95** | 0.96 | 1.05 | 42.32** | 55.91** | | Orbitofrontal cortex (superior), Left | Paralimbic | 1.35** | 1.45** | 0.94 | 1.29 | 1.07** | 0.82** | 57.68** | 29.22** | | Orbitofrontal cortex (superior), Right | Paralimbic | 1.38** | 1.41** | 1.00 | 1.17 | 1.01 | 0.88 | 49.86 | 34.74 | | Superior frontal gyrus, Right | Association | 1.31** | 1.43** | 0.90 | 0.92 | 1.10 | 1.08 | 61.45 | 59.70 | | Fusiform gyrus, Left | Association | 1.41 | 1.37 | 0.91 | 0.91 | 1.10 | 1.09 | 61.50 | 60.91 | | Fusiform gyrus, Right | Association | 1.42 | 1.32 | 0.90 | 0.87 | 1.11 | 1.12 | 62.82 | 65.39 | | Heschl's gyrus, Left | Primary | 1.60 | 1.62 | 1.10 | 1.12 | 0.93 | 0.91 | 40.32 | 37.00 | | Heschl's gyrus, Right | Primary | 1.57 | 1.65 | 1.10 | 1.13 | 0.93 | 0.90 | 39.59 | 36.17 | | Hippocampus, Left | Limbic | 1.41** | 1.50** | 1.03 | 1.04 | 0.98 | 0.97 | 46.14 | 45.09 | | Hippocampus, Right | Limbic | 1.40** | 1.48** | 1.00 | 1.01 | 1.01 | 1.00 | 49.36 | 48.57 | | Insula, Left | Paralimbic | 1.42 | 1.50 | 1.03 | 1.02 | 0.98 | 0.99 | 46.18 | 47.35 | | Insula, Right | Paralimbic | 1.42 | 1.52 | 1.04 | 1.02 | 0.97 | 0.99 | 44.27 | 48.30 | | Lingual gyrus, Left | Association | 1.47 | 1.41 | 0.92 | 0.92 | 1.08 | 1.08 | 59.91 | 60.30 | | Lingual gyrus, Right | Association | 1.45 | 1.40 | 0.91 | 0.91 | 1.09 | 1.09 | 60.95 | 61.39 | | Inferior occipital gyrus, Left | Association | 1.59 | 1.48 | 1.07** | 0.98** | 0.96** | 1.03** | 42.95** | 52.39** | | Inferior occipital gyrus, Right | Association | 1.58 | 1.45 | 1.06** | 0.97** | 0.97** | 1.04** | 45.73** | 53.96** | | Middle occipital gyrus, Left | Association | 1.44 | 1.41 | 0.92 | 0.91 | 1.08 | 1.09 | 59.73 | 61.70 | | Middle occipital gyrus, Right | Association | 1.46 | 1.48 | 0.95 | 0.98 | 1.06 | 1.03 | 56.05 | 52.26 | | Superior occipital gyrus, Left | Association | 1.51 | 1.50 | 0.96 | 0.98 | 1.04 | 1.03 | 54.45 | 53.00 | | Superior occipital gyrus, Right | Association | 1.49 | 1.50 | 0.99 | 0.99 | 1.03 | 1.02 | 52.00 | 51.09 | | Olfactory, Left | Limbic | 1.30 | 1.01 | 1.28 | 1.03 | 0.81 | 0.70 | 26.55 | 22.57 | | Olfactory, Right | Limbic | 1.16 | 0.84 | 1.28 | 1.00 | 0.79 | 0.63 | 23.73 | 18.52 | | Pallidum, Left | Subcortical | 1.43 | 1.54 | 1.17 | 1.16 | 0.88 | 0.89 | 33.73 | 34.96 | | Pallidum, Right | Subcortical | 1.25 | 1.41 | 1.17 | 1.16 | 0.88 | 0.88 | 34.59 | 34.70 | | Paracentral lobule, Left | Association | 1.48 | 1.54 | 0.95 | 1.05 | 1.06 | 0.97 | 55.86 | 44.65 | | Paracentral lobule, Right | Association | 1.43 | 1.54 | 0.93 | 1.05 | 1.07 | 0.96 | 57.95 | 44.35 | | Parahippocampus gyrus, Left | Paralimbic | 1.52 | 1.57 | 1.09 | 1.16 | 0.93 | 0.87 | 38.77 | 32.26 | | Parahippocampus gyrus, Right | Paralimbic | 1.47 | 1.45 | 0.99 | 1.04 | 1.02 | 0.97 | 51.59 | 45.30 | | Inferior parietal lobule, Left | Association | 1.45 | 1.49 | 1.03 | 1.03 | 0.98 | 0.98 | 45.64 | 46.13 | | Inferior parietal lobule, Right | Association | 1.50 | 1.54 | 1.14 | 1.13 | 0.89 | 0.89 | 34.91 | 34.78 | | Superior parietal gyrus, Left | Association | 1.45** | 1.52** | 0.95 | 1.09 | 1.06** | 0.93** | 55.91** | 40.09** | | Superior parietal gyrus, Right | Association | 1.44** | 1.58** | 0.96 | 1.14 | 1.05** | 0.89** | 55.50** | 34.52** | | Postcentral gyrus, Left | Primary | 1.51 | 1.47 | 0.98 | 0.97 | 1.03 | 1.03 | 52.86 | 53.74 | | Postcentral gyrus, Right | Primary | 1.51 | 1.49 | 0.99 | 0.99 | 1.03 | 1.02 | 52.41 | 52.30 | | Precentral gyrus, Left | Primary | 1.44 | 1.36 | 0.93** | 0.87** | 1.08 | 1.12 | 59.27 | 65.61 | | Precentral gyrus, Right | Primary | 1.46 | 1.35 | 0.95** | 0.87** | 1.07 | 1.13 | 57.50 | 66.17 | | Precuneus, Left | Association | 1.35 | 1.43 | 0.89 | 0.96 | 1.11 | 1.04 | 63.18 | 54.30 | | Precuneus, Right | Association | 1.37 | 1.47 | 0.92 | 0.99 | 1.08 | 1.02 | 59.55 | 51.26 | | Putamen, Left | Subcortical | 1.32** | 1.47** | 1.18** | 1.01** | 0.88** | 1.00** | 34.86** | 48.48** | | Putamen, Right | Subcortical | 1.43** | 1.48** | 1.22** | 1.02** | 0.87** | 0.99** | 34.91** | 47.48** | | Rectus gyrus, Left | Paralimbic | 1.47** | 1.51** | 1.05 | 1.36 | 0.97** | 0.76** | 45.45** | 21.17** | | Rectus gyrus, Right | Paralimbic | 1.49 | 1.47 | 1.09 | 1.35 | 0.94 | 0.77 | 40.86 | 21.61 | | Rolandic operculum, Left | Association | 1.57 | 1.62 | 1.07 | 1.10 | 0.95 | 0.93 | 42.59 | 39.48 | | Rolandic operculum, Right | Association | 1.54 | 1.63 | 1.05 | 1.10 | 0.97 | 0.92 | 45.14 | 38.65 | | Supplementary motor area, Left | Association | 1.35 | 1.37 | 0.91 | 0.88 | 1.10 | 1.12 | 61.64 | 64.87 | | Supplementary motor area, Right | Association | 1.32 | 1.37 | 0.89 | 0.88 | 1.11 | 1.12 | 63.00 | 64.91 | | Supramarginal gyrus, Left | Association | 1.53 | 1.56 | 1.06** | 1.07** | 0.96 | 0.95 | 43.59** | 42.61** | | Supramarginal gyrus, Right | Association | 1.48 | 1.53 | 1.08** | 1.03** | 0.95 | 0.98 | 41.41** | 46.13** | | Inferior temporal gyrus, Left | Association | 1.35 | 1.37 | 0.89 | 0.94 | 1.11 | 1.06 | 63.82 | 57.43 | | Inferior temporal gyrus, Right | Association | 1.31 | 1.33 | 0.84 | 0.89 | 1.16 | 1.11 | 69.68 | 63.91 | | Middle temporal gyrus, Left | Association | 1.43 | 1.39 | 0.95 | 0.89 | 1.06 | 1.11 | 56.41 | 63.35 | | Middle temporal gyrus, Right | Association | 1.39 | 1.35 | 0.89 | 0.85 | 1.11 | 1.14 | 63.23 | 67.43 | | Temporal pole (middle), Left | Paralimbic | 1.59 | 1.13 | 1.08** | 1.18** | 0.95 | 0.79 | 41.95 | 27.22 | | Temporal pole (middle), Right | Paralimbic | 1.54 | 1.39 | 1.02** | 1.16** | 1.00 | 0.87 | 48.41 | 32.39 | | Temporal pole (superior), Left | Paralimbic | 1.42 | 1.34 | 0.94 | 1.05 | 1.07 | 0.97 | 57.36 | 46.35 | | Temporal pole (superior), Right | Paralimbic | 1.35 | 1.40 | 0.91 | 0.99 | 1.09 | 1.03 | 60.18 | 52.65 | | Superior temporal gyrus, Left | Association | 1.50 | 1.51 | 1.00 | 0.99 | 1.01 | 1.02 | 51.00 | 51.91 | | Superior temporal gyrus, Right | Association | 1.46 | 1.46 | 0.96 | 0.94 | 1.04 | 1.06 | 54.36 | 56.35 | | Thalamus, Left | Subcortical | 1.39** | 1.41** | 1.10** | 0.94** | 0.95** | 1.06** | 44.36** | 57.61** | | Thalamus, Right | Subcortical | 1.40** | 1.41** | 1.11** | 0.94** | 0.95** | 1.06** | 43.77** | 57.57** | |
| --- | --- | --- | --- | --- | --- | --- | --- | --- | --- | --- | --- | --- | --- | --- | --- | --- | --- | --- | --- | --- | --- | --- | --- | --- | --- | --- | --- | --- | --- | --- | --- | --- | --- | --- | --- | --- | --- | --- | --- | --- | --- | --- | --- | --- | --- | --- | --- | --- | --- | --- | --- | --- | --- | --- | --- | --- | --- | --- | --- | --- | --- | --- | --- | --- | --- | --- | --- | --- | --- | --- | --- | --- | --- | --- | --- | --- | --- | --- | --- | --- | --- | --- | --- | --- | --- | --- | --- | --- | --- | --- | --- | --- | --- | --- | --- | --- | --- | --- | --- | --- | --- | --- | --- | --- | --- | --- | --- | --- | --- | --- | --- | --- | --- | --- | --- | --- | --- | --- | --- | --- | --- | --- | --- | --- | --- | --- | --- | --- | --- | --- | --- | --- | --- | --- | --- | --- | --- | --- | --- | --- | --- | --- | --- | --- | --- | --- | --- | --- | --- | --- | --- | --- | --- | --- | --- | --- | --- | --- | --- | --- | --- | --- | --- | --- | --- | --- | --- | --- | --- | --- | --- | --- | --- | --- | --- | --- | --- | --- | --- | --- | --- | --- | --- | --- | --- | --- | --- | --- | --- | --- | --- | --- | --- | --- | --- | --- | --- | --- | --- | --- | --- | --- | --- | --- | --- | --- | --- | --- | --- | --- | --- | --- | --- | --- | --- | --- | --- | --- | --- | --- | --- | --- | --- | --- | --- | --- | --- | --- | --- | --- | --- | --- | --- | --- | --- | --- | --- | --- | --- | --- | --- | --- | --- | --- | --- | --- | --- | --- | --- | --- | --- | --- | --- | --- | --- | --- | --- | --- | --- | --- | --- | --- | --- | --- | --- | --- | --- | --- | --- | --- | --- | --- | --- | --- | --- | --- | --- | --- | --- | --- | --- | --- | --- | --- | --- | --- | --- | --- | --- | --- | --- | --- | --- | --- | --- | --- | --- | --- | --- | --- | --- | --- | --- | --- | --- | --- | --- | --- | --- | --- | --- | --- | --- | --- | --- | --- | --- | --- | --- | --- | --- | --- | --- | --- | --- | --- | --- | --- | --- | --- | --- | --- | --- | --- | --- | --- | --- | --- | --- | --- | --- | --- | --- | --- | --- | --- | --- | --- | --- | --- | --- | --- | --- | --- | --- | --- | --- | --- | --- | --- | --- | --- | --- | --- | --- | --- | --- | --- | --- | --- | --- | --- | --- | --- | --- | --- | --- | --- | --- | --- | --- | --- | --- | --- | --- | --- | --- | --- | --- | --- | --- | --- | --- | --- | --- | --- | --- | --- | --- | --- | --- | --- | --- | --- | --- | --- | --- | --- | --- | --- | --- | --- | --- | --- | --- | --- | --- | --- | --- | --- | --- | --- | --- | --- | --- | --- | --- | --- | --- | --- | --- | --- | --- | --- | --- | --- | --- | --- | --- | --- | --- | --- | --- | --- | --- | --- | --- | --- | --- | --- | --- | --- | --- | --- | --- | --- | --- | --- | --- | --- | --- | --- | --- | --- | --- | --- | --- | --- | --- | --- | --- | --- | --- | --- | --- | --- | --- | --- | --- | --- | --- | --- | --- | --- | --- | --- | --- | --- | --- | --- | --- | --- | --- | --- | --- | --- | --- | --- | --- | --- | --- | --- | --- | --- | --- | --- | --- | --- | --- | --- | --- | --- | --- | --- | --- | --- | --- | --- | --- | --- | --- | --- | --- | --- | --- | --- | --- | --- | --- | --- | --- | --- | --- | --- | --- | --- | --- | --- | --- | --- | --- | --- | --- | --- | --- | --- | --- | --- | --- | --- | --- | --- | --- | --- | --- | --- | --- | --- | --- | --- | --- | --- | --- | --- | --- | --- | --- | --- | --- | --- | --- | --- | --- | --- | --- | --- | --- | --- | --- | --- | --- | --- | --- | --- | --- | --- | --- | --- | --- | --- | --- | --- | --- | --- | --- | --- | --- | --- | --- | --- | --- | --- | --- | --- | --- | --- | --- | --- | --- | --- | --- | --- | --- | --- | --- | --- | --- | --- | --- | --- | --- | --- | --- | --- | --- | --- | --- | --- | --- | --- | --- | --- | --- | --- | --- | --- | --- | --- | --- | --- | --- | --- | --- | --- | --- | --- | --- | --- | --- | --- | --- | --- | --- | --- | --- | --- | --- | --- | --- | --- | --- | --- | --- | --- | --- | --- | --- | --- | --- | --- | --- | --- | --- | --- | --- | --- | --- | --- | --- | --- | --- | --- | --- | --- | --- | --- | --- | --- | --- | --- | --- | --- | --- | --- | --- | --- | --- | --- | --- | --- | --- | --- | --- | --- | --- | --- | --- | --- | --- | --- | --- | --- | --- | --- | --- | --- | --- | --- | --- | --- | --- | --- | --- | --- | --- | --- | --- | --- | --- | --- | --- | --- | --- | --- | --- | --- | --- | --- | --- | --- | --- | --- | --- | --- | --- | --- | --- | --- | --- | --- | --- | --- | --- | --- | --- | --- | --- | --- | --- | --- | --- | --- | --- | --- | --- | --- | --- | --- | --- | --- | --- | --- | --- | --- | --- | --- | --- | --- | --- | --- | --- | --- | --- | --- | --- | --- | --- | --- | --- | --- | --- | --- | --- | --- | --- | --- | --- | --- | --- | --- | --- | --- | --- | --- | --- | --- | --- | --- | --- | --- | --- | --- | --- | --- | --- | --- | --- | --- | --- | --- | --- | --- | --- | --- | --- | --- | --- | --- | --- | --- | --- | --- | --- | --- | --- | --- | --- | --- | --- | --- | --- | --- | --- | --- | --- | --- | --- | --- | --- | --- | --- | --- | --- | --- | --- | --- | --- | --- | --- | --- | --- | --- | --- | --- | --- | --- | --- | --- | --- | --- | --- | --- | --- | --- | --- | --- | --- | --- | --- | --- | --- | --- | --- | --- | --- | --- | --- | --- | --- | --- | --- | --- | --- | --- | --- | --- | --- | --- | --- | --- | --- | --- | --- | --- | --- | --- | --- | --- | --- | --- |

Graph metrics showing normalized clustering coefficient (γ_i_), normalized path length (λ_i_), normalized efficiency (Eglobal_i_), and degree (K_i_), of the ith region. Each region was classified as subcortical or primary, association, limbic, or paralimbic cortex based on the parcellation scheme proposed by Mesulam [1] . Graph metrics which were significantly different (p < 0.01) in children, compared to young-adults, are indicated in **. The p-value was computed by comparing the fitted growth curve of each metric (γ_i_, λ_i_, Eglobal_i_,K_i_) for a range of threshold values from 0.1 to 0.6.
