# Supplementary material for: Development of Large-Scale Functional Brain Networks in Children
Source: PLoS Biol. 2009 Jul 21;7(7):e1000157. doi: 10.1371/journal.pbio.1000157 (PMC2705656; doi:10.1371/journal.pbio.1000157)
Supplement: Text S1 — Experimental procedures. (0.07 MB DOC) [file pbio.1000157.s003.doc]

**Supplementary Text S1**

**Experimental Procedures**

**Classification analysis of whole-brain functional connectivity patterns in children and young-adults**

To investigate the developmental differences in whole-brain functional connectivity patterns, we examined the patterns in the two groups. The functional connectivity patterns -- the correlation values of 4005 pairs of anatomical regions – were used as the input (features) to a pattern-based classifier. The classifier distinguishes young-adults from children by making classification decision based on value of the linear combination of these features. A widely used linear-classifier (Support Vector Machine Classifier) that was best suited for our purpose of classification based on large number of features (4005) but a small number of training samples (45), was used in our analysis. Leave-one-out cross-validation (LOOCV) was used to measure the performance of the classifier in distinguishing young-adults from children. In LOOCV, one single observation is used for testing the classifier that is trained using the remaining observations. This process is repeated such that every observation is used once for testing purposes. This analysis was performed using the WEKA software, which is a java based package for pattern recognition (<http://www.cs.waikato.ac.nz/ml/weka/>).

**Classification analysis of inter-regional functional connectivity patterns in children and young-adults**

The inter-regional connectivity patterns – the correlation values between five major divisions of the brain – were used as features for a Support vector classifier. LOOCV accuracy values for each of the 10 pairs of divisions were computed, as described above.

**Anatomical distance between brain regions.**

The anatomical distance between brain regions was computed by measuring the average length of the fiber tracks connecting those regions. Fiber tracks were derived using diffusion tensor imaging (DTI) data obtained from 18 of the 23 children subjects and 15 of 22 young-adults.

The DTI pulse sequence was a diffusion-weighted single-shot spin-echo, echo planar imaging sequence (TE = 70.8 ms; TR = 8.6 s; field of view = 220 mm; matrix size = 128 x 128; bandwidth = ±110kHz; partial k-space acquisition). We acquired 63 axial, 2-mm thick slices (no skip) for 2 *b* values, b = 0 and b = approximately 850 s/mm^2^. The high b value was obtained by applying gradients along 46 different diffusion directions (23 noncollinear directions). Two gradient axes were energized simultaneously to minimize TE. The polarity of the effective diffusion-weighting gradients was reversed for odd repetitions to reduce cross-terms between diffusion gradients and imaging and background gradients. Although Jones [2] suggests that measuring more diffusion directions would be a more efficient way to reliably estimate diffusion tensors of arbitrary orientation, our signal-to-noise ratio is sufficiently high from our 4 repeats to produce very reliable tensor estimates suitable for tractography.

DTI data were preprocessed using a custom program based on normalized mutual information that removed eddy current distortion effects and determined a constrained nonrigid image registration of all the diffusion images [3]. The 6 elements of the diffusion tensor were determined by multivariate regression [4,5]. For each subject, the non–diffusion-weighted (*b* = 0) images were coregistered to the T1-weighted 3-D SPGR anatomical images using a mutual information 3-D rigid-body coregistration algorithm from SPM2. Several anatomical landmarks, including the anterior commissure (AC), the posterior commissure (PC), and the midsagittal plane, were identified by hand in the T1 images. With these landmarks, we computed a rigid-body transform from the native image space to the conventional AC-PC–aligned space. The DTI data were then resampled to this AC-PC–aligned space with 2-mm isotropic voxels using a spline-based tensor interpolation algorithm [6], taking care to rotate the tensors to preserve their orientation with respect to the anatomy [7]. The T1 images were resampled to AC-PC–aligned space with 1-mm isotropic voxels. We confirmed by visual inspection of each dataset that this coregistration technique aligns the DTI and T1 images to within 1–2 millimeters in the brain regions of interest.

The 90 anatomical regions of interest, which were in the MNI space, were warped back to each individual brain so that they could be used for subsequent DTI tractography analyses. This was done by applying the inverse of the spatial normalization transformation. As fiber tracking becomes unreliable in gray matter, we ensured that our ROIs extended 2–3mm into the white matter.

DTI fiber tractography was used to estimate the likely connections between the 90 anatomical regions of interest. Using custom DTI analysis software (available for download at <http://sirl.stanford.edu/software/>), the tractography procedure was initiated by whole-brain fiber tracking that produced many fiber paths. Tracts that did not end in both ROIs were discarded. Each fiber tract was estimated using a deterministic streamlines tracking algorithm [8,9,10] with a fourth order Runge-Kutta path integration method and 1 mm fixed step size. A continuous tensor field was estimated using trilinear interpolation of the tensor elements. Starting from the initial seed point, fiber paths were traced in both directions along the principal diffusion axis. Path tracing proceeded until the FA fell below 0.15 or until the minimum angle between the current and previous path segments was larger than 30°.

To combine resulting fiber maps, individual tracts were translated into a common space by applying the spatial normalization parameters that map each individual brain to the same MNI template to which the fMRI data were aligned. The deformation field resulting from the spatial normalization calculation was applied to the fiber coordinates from each individual brain to bring them all into a common space. The anatomical distance between two brain regions was obtained by computing the average of length of fiber tracts, in the common MNI space, connecting those regions, averaged across all the subjects. The fiber length was computed in MNI space, instead of native space, to rule out the effect of age-related changes in inter-regional fiber length on our findings.

**Analysis of functional connectivity matrices**

Group averaged 90-by-90 correlation matrices for young-adults and children are shown in Figure 5. Value of the *(i,j)*th element of the correlation matrix corresponds to group averaged scale 3 wavelet correlation between the resting-state timeseries of brain region *i* and region *j*. Low correlation values are shown in darker color while high correlation values are shown in lighter color.

Inter-subject variability in these correlation values was low in both the groups. Specifically, correlation values in the young-adult group showed inter-subject variance of 0.046 while the correlation values in the child group showed inter-subject variance of 0.036. Between group comparison revealed higher inter-subject variance in the young-adults compared to children (p < 0.01).

A detailed description of quantitative between group differences observed in these correlation matrices is presented in the results section as well as in Figure 4

**References**

1. Mesulam MM (2000) Principles of behavioral and cognitive neurology. 2nd ed. Oxford: Oxford University Press. pp. xviii, 540 p.

2. Jones DK (2004) The effect of gradient sampling schemes on measures derived from diffusion tensor MRI: a Monte Carlo study. Magn Reson Med 51: 807-815.

3. Bammer R, Auer M, Keeling SL, Augustin M, Stables LA, et al. (2002) Diffusion tensor imaging using single-shot SENSE-EPI. Magn Reson Med 48: 128-136.

4. Basser PJ (1995) Inferring microstructural features and the physiological state of tissues from diffusion-weighted images. NMR Biomed 8: 333-344.

5. Basser PJ, Pierpaoli C (1996) Microstructural and physiological features of tissues elucidated by quantitative-diffusion-tensor MRI. J Magn Reson B 111: 209-219.

6. Pajevic S, Aldroubi A, Basser PJ (2002) A continuous tensor field approximation of discrete DT-MRI data for extracting microstructural and architectural features of tissue. J Magn Reson 154: 85-100.

7. Alexander DC, Pierpaoli C, Basser PJ, Gee JC (2001) Spatial transformations of diffusion tensor magnetic resonance images. IEEE Trans Med Imaging 20: 1131-1139.

8. Conturo TE, Lori NF, Cull TS, Akbudak E, Snyder AZ, et al. (1999) Tracking neuronal fiber pathways in the living human brain. Proc Natl Acad Sci U S A 96: 10422-10427.

9. Mori S, Crain BJ, Chacko VP, van Zijl PC (1999) Three-dimensional tracking of axonal projections in the brain by magnetic resonance imaging. Ann Neurol 45: 265-269.

10. Basser PJ, Pajevic S, Pierpaoli C, Duda J, Aldroubi A (2000) In vivo fiber tractography using DT-MRI data. Magn Reson Med 44: 625-632.
